# Neurogenic Effect of Hypoxia-Conditioned UC-MSC Secretome from Macaca Fascicularis in an *in Vitro* Stroke Model

**DOI:** 10.34172/apb.46137

**Published:** 2026-05-19

**Authors:** Gita Serafika Shannon, Ratih Rinendyaputri, Sela Septima Mariya, Sunarno Sunarno, Amarila Malik, Rachmawati Noverina, Putri Retno Intan, Wireni Ayuningtyas, Fathul Huda, Ahmad Faried

**Affiliations:** ^1^Graduate School of Pharmaceutical Sciences, Faculty of Pharmacy, Universitas Indonesia, Depok 16424, Indonesia; ^2^Center for Biomedical Research, Research Organization for Health, National Research and Innovation Agency, Bogor, Indonesia 16911; ^3^Division of Pharmaceutical Microbiology and Biotechnology, Faculty of Pharmacy Universitas Indonesia, Depok 16424, Indonesia; ^4^Bio Farma Stem Cell Research and Development, Bandung, Indonesia 40161; ^5^Department of Neurosurgery, Faculty of Medicine, Universitas Padjadjaran, Bandung, Indonesia 40161

**Keywords:** Ischemic stroke, Neurogenesis, Oxygen glucose deprivation (OGD), Secretome, SHSY-5Y

## Abstract

**Purpose:**

Mesenchymal stem cells (MSCs) hold therapeutic promise for ischemic stroke through paracrine release of bioactive factors that promote angiogenesis and neurogenesis. This study investigated the neurogenic potential of secretomes derived from Macaca fascicularis UC-MSCs cultured under hypoxic and normoxic conditions in an in vitro ischemic stroke model and evaluated their potential as a regenerative, cell-free therapeutic strategy.

**Methods:**

mUC-MSCs were cultured under normoxic (21% O2) and hypoxic (3% O2) conditions for 48 hours. Secretomes were collected and analyzed for Brain-Derived Neurotrophic Factor (BDNF) and Stromal Cell-Derived Factor-1 (SDF-1) levels via enzyme-linked immunosorbent assay (ELISA), while exosome morphology was characterized using scanning electron microscopy (SEM). An in vitro ischemic stroke model was created using SH-SY5Y cells exposed to 6 hours of oxygen-glucose deprivation (OGD) followed by 24 hours of reperfusion. During reperfusion, cells were treated with normoxic or hypoxic secretome. Neurogenesis-related gene expression was assessed by RT-PCR and statistically analyzed.

**Results:**

The secretome from 3% O2 hypoxic preconditioning showed elevated levels of BDNF and SDF-1 compared to normoxic conditions. RT-qPCR analysis revealed significant upregulation of neurogenesis-related genes BDNF, TrkB, ERK1/2, Nestin, and β-tubulin following treatment with the hypoxic secretome, indicating enhanced neurogenic activity and neuronal survival in the in vitro ischemic stroke model.

**Conclusion:**

The hypoxia-preconditioned UC-MSC secretome enhances neurogenesis by upregulating key neurogenic markers, promoting neuronal differentiation and regeneration under ischemic conditions. These findings highlight the promise of this approach as a regenerative, cell-free therapy for promoting recovery following ischemic stroke.

## Introduction

 Stroke is the second leading cause of mortality and a major cause of long-term disability, particularly in low- and middle-income countries. Ischemic stroke constitutes approximately 65% of cases, followed by intracerebral hemorrhage (29%) and subarachnoid hemorrhage (6%).^1,2^ Annually, stroke affects around 11.71 million people globally, with incidence expected to more than double by 2050.^1^ While recombinant tissue plasminogen activator (rtPA) is the standard treatment for acute ischemic stroke, its use is limited to about 35% of eligible patients due to a narrow therapeutic window, bleeding risk, and various contraindications.^3^ These limitations underscore the urgent need for alternative or adjunctive therapies. Neuroprotective strategies, which aim to preserve the ischemic penumbra by modulating molecular and cellular pathways, show promise but have yet to achieve consistent success in clinical translation.^4^

 Stem cell-based therapies represent a promising strategy for stroke recovery by promoting neural repair through multiple biological pathways. Mesenchymal Stem Cells (MSCs), particularly, have attracted attention for their potent paracrine activity, surpassing their differentiation capacity.^5,6^ MSCs release a wide range of bioactive molecules such as vascular endothelial growth factor (VEGF), insulin-like growth factor-1 (IGF-1), basic fibroblast growth factor (bFGF), transforming growth factor-β1 (TGF-β1), and nerve growth factor (NGF), and cytokines including stromal cell-derived factor-1 (SDF-1/CXCL12), monocyte chemoattractant protein-1 (MCP-1/CCL2), and interleukins IL-6, IL-8, IL-10, and IL-13 which collectively promote neuroprotection, angiogenesis, immunomodulation, and neurogenesis.^7–9^ Hypoxic preconditioning has been shown to boost the secretion of these regenerative factors, thereby improving the therapeutic potential of MSC-derived secretomes.^10^ The MSC secretome plays a central role in tissue repair and is increasingly recognized as the main mediator of therapeutic effects in ischemic stroke, offering a cell-free approach to stem cell transplantation.^11,12^

 In vitro stroke models, particularly the oxygen-glucose deprivation (OGD) model, provide a controlled and reproducible platform to study ischemic mechanisms and evaluate neuroprotective strategies.^13,14^ These models enable detailed investigation of cellular responses within the ischemic penumbra and are valuable for preclinical drug screening. Experimental evidence indicates that bone marrow–derived MSCs in rodent stroke models reduce neuroinflammation and enhance neurogenesis, in part by redirecting microglia and macrophages from a pro-inflammatory M1 phenotype toward a reparative M2 state.^15^

 Leveraging advances in cell-free regenerative therapies, the secretome of *Macaca fascicularis* umbilical cord-derived MSCs (UC-MSCs) offers a novel therapeutic avenue focan r stroke recovery. As a non-human primate, M. fascicularis offers greater translational relevance than rodent models.^16,17^ While human MSC secretomes show therapeutic promise, their scalability remains limited by production costs. Primate-derived secretomes offer a more accessible and ethically acceptable alternative for preclinical studies. While secretome profiles of M. fascicularis UC-MSCs under normoxia and hypoxia are known, their effects on post-ischemic neurogenesis remain unclear.^18^ To address this, the regenerative potential of normoxia- and hypoxia-preconditioned UC-MSC secretomes was evaluated using SH-SY5Y cells subjected to OGD, assessing their suitability as a cell-free therapeutic strategy for ischemic stroke recovery.

## Materials and Methods

###  Collection and Isolation of Macaca Fascicularis UCMSCs

 Umbilical cord MSCs were isolated from *Macaca fascicularis* fetuses obtained ethically from PT Bio Farma (No. 224K-Mon-Nef01). Umbilical cord tissues collected by caesarean section at approximately 120 days gestation were processed and cultured in MEM supplemented with 10–20% FBS and 1% penicillin–streptomycin. Cells were incubated at 37 °C, 5% CO_2_, with medium replaced every two days.^19^

###  Phenotypic and Differentiation Characterization of UCMSCs

 UC-MSCs were characterized phenotypically by flow cytometry using a BD Biosciences antibody panel and analyzed on a BD Accuri^TM^ C6 Plus flow cytometer to assess surface marker expression of positive markers (CD73, CD90, CD105) and the absence of negative markers (CD34, CD45).^19^ Multipotency was evaluated using the StemPro^TM^ differentiation kit (Gibco), assessing the capacity for adipogenic, osteogenic, and chondrogenic lineage differentiation.^20^

###  Hypoxia Preconditioning and Secretome Collection

 Hypoxic preconditioning was conducted in a two-gas incubator (Thermo Scientific) set at 3% O_2_, 5% CO_2_, and 37 °C. Upon reaching ~80% confluency, UC-MSCs were cultured in serum-free MEM and exposed to hypoxia for 48 hours. Conditioned medium was collected, centrifuged at 1,200 rpm for 10 minutes to remove debris, and then filtered through a 0.22 µm syringe filter.^19^

###  Quantification of BDNF and SDF-1 and Statistical Analysis

 BDNF and SDF-1 levels were quantified using ELISA kits (BDNF: ELK Biotechnology, ELK1394; SDF-1: Bioenzy, BZ-08123533-EB) according to the manufacturers’ instructions.^19^ All experiments were conducted using three independent biological replicates, which were subsequently pooled into a single sample, and each measurement was performed in triplicate technical repeats. Absorbance was measured at 450 nm using a Bio-Rad microplate reader, and concentrations were calculated from standard curves. Statistical analysis was performed using IBM SPSS Statistics v23.0. Independent Student’s t-tests were applied for single comparison for protein levels between hypoxia- and normoxia-treated groups. A p-value < 0.05 was considered statistically significant.

###  Scanning Electron Microscopy (SEM) 

 Exosome isolation was performed through a series of differential centrifugation steps. Initially, conditioned media were centrifuged at 300 × g for 10 minutes at 4 °C to eliminate dead cells, followed by centrifugation at 2,000 × g for 30 minutes and subsequently at 21,000 × g for 90 minutes to remove cellular debris and larger extracellular vesicles. Pellets were resuspended in PBS and ultracentrifuged at 100,000 × g for 70 min to isolate exosomes. Isolates were stored at −80 °C until further use.^21^ Exosome isolates from three independent biological replicates were pooled prior to SEM analysis.

###  SH-SY5Y Cell Culture and Subculturing Protocol

 SH-SY5Y human neuroblastoma cells (ATCC) were cultured in Dulbecco’s Modified Eagle Medium (DMEM) enriched with 10% FBS, 1% L-glutamine, and 1% penicillin–streptomycin. After thawing, cells were centrifuged at 300 × g for 5 min and viability assessed by trypan blue exclusion. Cells were cultured at a density of 1 × 10^4^ cells/cm^2^ in 6-well plates containing 2 mL of complete medium per well and incubated at 37 °C in a humidified atmosphere with 5% CO_2_. The medium was replenished every 48 hours. Upon reaching 70–80% confluency, cells were passaged using 0.05% trypsin–EDTA and reseeded until a final density of approximately 1.2 × 10^6^ cells per well was achieved.

###  Immunocytochemistry

 Immunocytochemistry was performed to assess nestin expression, following a previously described protocol.^22^ Cells were fixed with 4% paraformaldehyde (15 min), washed with PBS, and treated with 3% hydrogen peroxide in methanol (15 min) to quench endogenous peroxidase activity. Non-specific binding was blocked with Background Sniper (Starr Trek Universal HRP Detection Kit, Biocare; 15 min). An overnight incubation at 4 °C was performed using a primary antibody targeting nestin, followed by HRP-conjugated secondary antibody (Biocare) (15 min) and Trek-Avidin-HRP (15 min). Detection was performed using DAB substrate (1–2 min), and cells were counterstained with Mayer’s hematoxylin (1–2 min). Slides were rinsed and observed under a light microscope. Immunocytochemistry was performed on three independent biological replicates

###  Oxygen–Glucose Deprivation and Reperfusion (OGD/R) Model 

 To model ischemia, SH-SY5Y cells were exposed to OGD medium (20% low-glucose DMEM, 80% glucose-free DMEM) for 6 h at 37 °C under either normoxia (~21% O_2_) or hypoxia (3% O_2_). Controls were maintained in DMEM + 10% FBS under normoxia.

 Following 6 hours of oxygen-glucose deprivation (OGD) treatment, the culture medium was substituted with conditioned medium (CM) for subsequent analysis, and cells were cultured under normoxic reperfusion conditions (21% O_2_, 5% CO_2_, 37 °C) for 24 hours. Treatment groups included SH-SY5Y cells exposed to OGD and then treated with low-glucose DMEM containing 10% FBS and secretome from either hypoxia-preconditioned (3% O_2_) or normoxia-preconditioned *Macaca fascicularis* UCMSCs. Control cells were maintained in complete DMEM under normoxia, while the negative control consisted of OGD-exposed cells without post-treatment.The model is summarized in Table 1.

**Table 1:** An in vitro Oxygen-Glucose Deprivation/Reperfusion (OGD/R) model

**No**	**3% O2 for 6 hours (OGD) - DMEM low glucose and no glucose**	**Reperfusion therapy 21% O2 for 24 hours**
1	SH-SY5Y	DMEM *low glucose* 10% FBS
2	SH-SY5Y	Hypoxia-preconditioned mUCMSC secretome
3	SH-SY5Y	Normoxia-preconditioned mUCMSC secretome
4	SH-SY5Y	Without reperfusion (OGD SH-SY5Y)

###  RNA Isolation and Assessment of mRNA Expression via RT-Quantitative PCR (RT-qPCR)

 Total RNA was extracted using the RNA Simple Total RNA Kit (Tiangen, China) and quantified with a NanoDrop spectrophotometer (Thermo Scientific, USA). Samples were standardized to 10 ng/µL and reverse-transcribed into cDNA using the ReverTra Ace-α Kit (Toyobo, Japan). The cDNA was stored at –20 °C until use.

 Gene expression of BDNF, TrkB, ERK1/2, Nestin, β-tubulin, and ACTB was quantified by RT-qPCR with SsoFast EvaGreen Supermix (Bio-Rad) on an Applied Biosystems 7500 system. All experiments were conducted using three independent biological replicates, which were subsequently pooled into a single sample, and each measurement was performed in triplicate technical repeats. Reactions (20 µL) contained 2 µL cDNA, 10 µL Supermix, 1 µL each primer, and 6 µL water. Cycling conditions were 95 °C for 30 s, followed by 40 cycles of 98 °C for 5 s and 57 °C for 15 s. ACTB served as the internal control, and relative expression was calculated using the 2^–ΔCt method. Primer sequences are listed in Table 2.

**Table 2:** Sequences of Oligonucleotides Used in Polymerase Chain Reaction

**Number**	**Primer Used**	**Primer Sequence (5’-3’)**	**Reference**
1.	ACTB Forward ACTB Reverse	AGAGCTACGAGCTGCCTGAC AGCACTGTGTTGGCGTACAG	^23^
2.	Nestin Forward Nestin Reverse	GCGTTGGAACAGAGGTTGGA TGGGAGCAAAGATCCAAGAC	^24,25^
3.	B-tubulin Forward B-tubulin Reverse	CGAAGCCAGCAGTGTCTAAA GGAGGACGAGGCCATAAATAC	^26^
4.	ERK1/2 Forward ERK1/2 Reverse	ACAGGCTGTTCCCAAATGCT CGAACTTGAATGGTGCTTCG	^27^
5.	TrkB Forward TrkB Reverse	ACCCGAAACAAACTGACGAGT AGCATGTAAATGGATTGCCCA	^28^
6.	BDNF Forward BDNF Reverse	GGCTTGACATCATTGGCTGAC CATTGGGCCGAACTTTCTGGT	^28^

###  Statistical Analysis 

 RT-qPCR data were analyzed using IBM SPSS Statistics v23.0. Relative expression levels of BDNF, TrkB, ERK1/2, Nestin, and β-tubulin were calculated by the 2^−ΔCt method with ACTB as the reference gene.^23^ Group comparisons (normoxic secretome, hypoxic secretome, DMEM + 10% FBS, and OGD-only control) were performed using one-way ANOVA with Tukey’s post hoc test, or Kruskal–Wallis with Mann–Whitney U when assumptions of normality or homogeneity were not met. Results are expressed as mean ± SD, with significance set at *P*< 0.05.

## Results

###  Phenotypic Characterization and Differentiation Potential of UC-MSC Samples

*Macaca fascicularis* UC-MSCs at passage 5 were validated by flow cytometry, showing strong expression of MSC markers (CD73, CD90, CD105, CD44) and absence of hematopoietic/immune markers (CD34, CD11B, CD19, CD45, HLA-DR). (Figure 1).

**Figure 1:**
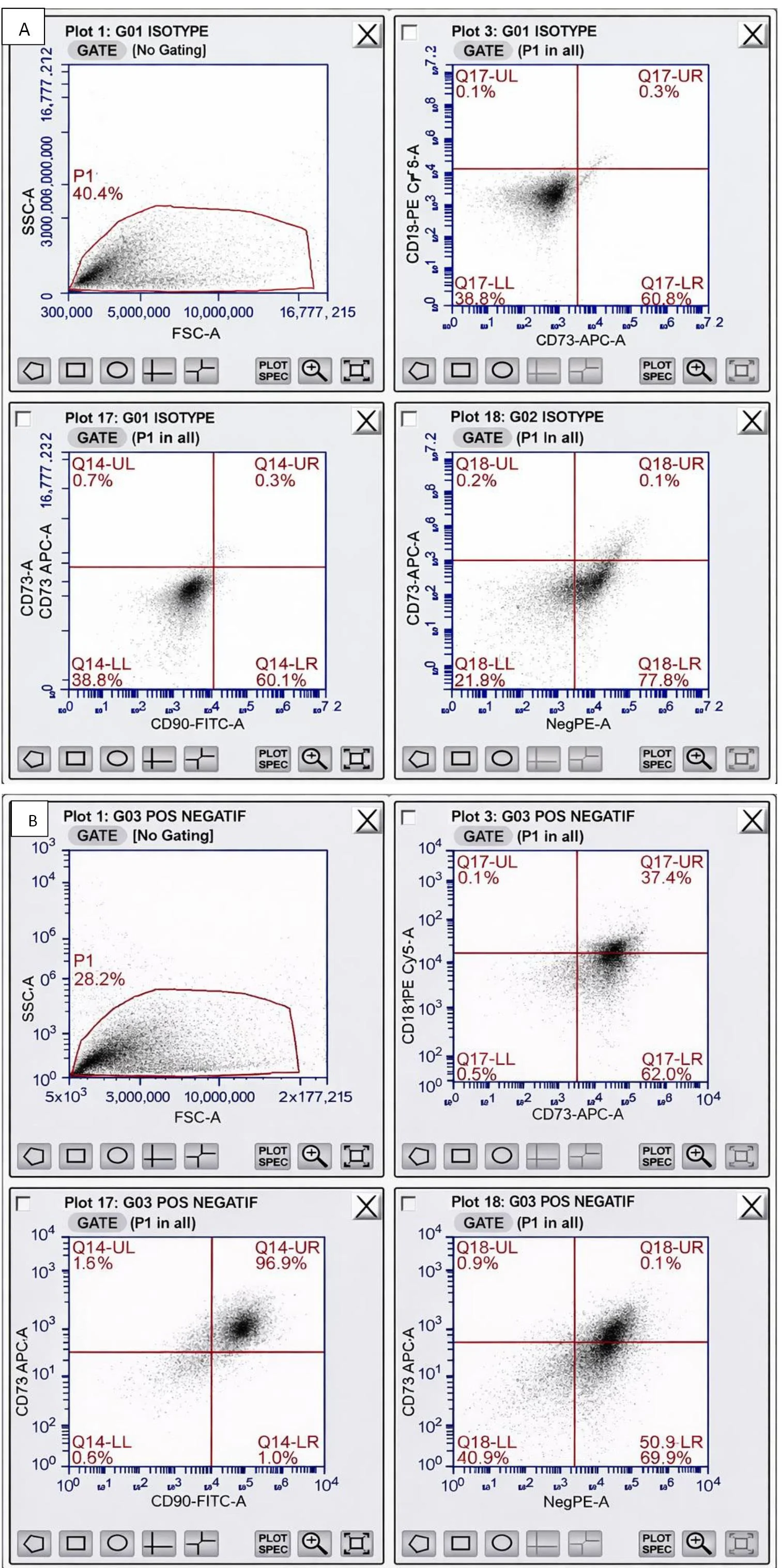
Flow cytometry characterization of UC-MSCs at passage 5. (A) Isotype control. (B) Expression of positive (CD73, CD90, CD105, CD44) and negative (CD34, CD45) surface markers confirming MSC phenotype

 BDNF and SDF-1 levels in the UC-MSC-derived secretome were measured using ELISA. Results showed that hypoxic preconditioning enhanced the secretion of both BDNF and SDF-1 by approximately 1.5-fold compared to normoxic conditions (Table 3).

**Table 3:** Concentrations of BDNF and SDF-1 in Hypoxia-Preconditioned UC-MSC-Derived Secretome

**Sampel**	**BDNF**		**SDF-1**	
	**Concentration Mean (pg/mL)**	***Std. Dev***	**Concentration Mean (pg/mL)**	***Std. Dev***
Normoxic	109.1289	0.8697	7,396.1004	1.4808
Hypoxic	155.1368	0.2057	11,222.2690	0.9181

 Comparative analysis revealed a significant upregulation of BDNF and SDF-1 protein levels in the secretome from hypoxia-preconditioned UC-MSCs compared to normoxic controls (Figure 2). This finding highlights the enhanced paracrine potential of hypoxia-primed MSCs, driven by elevated secretion of critical neurotrophic and chemotactic factors.

**Figure 2:**
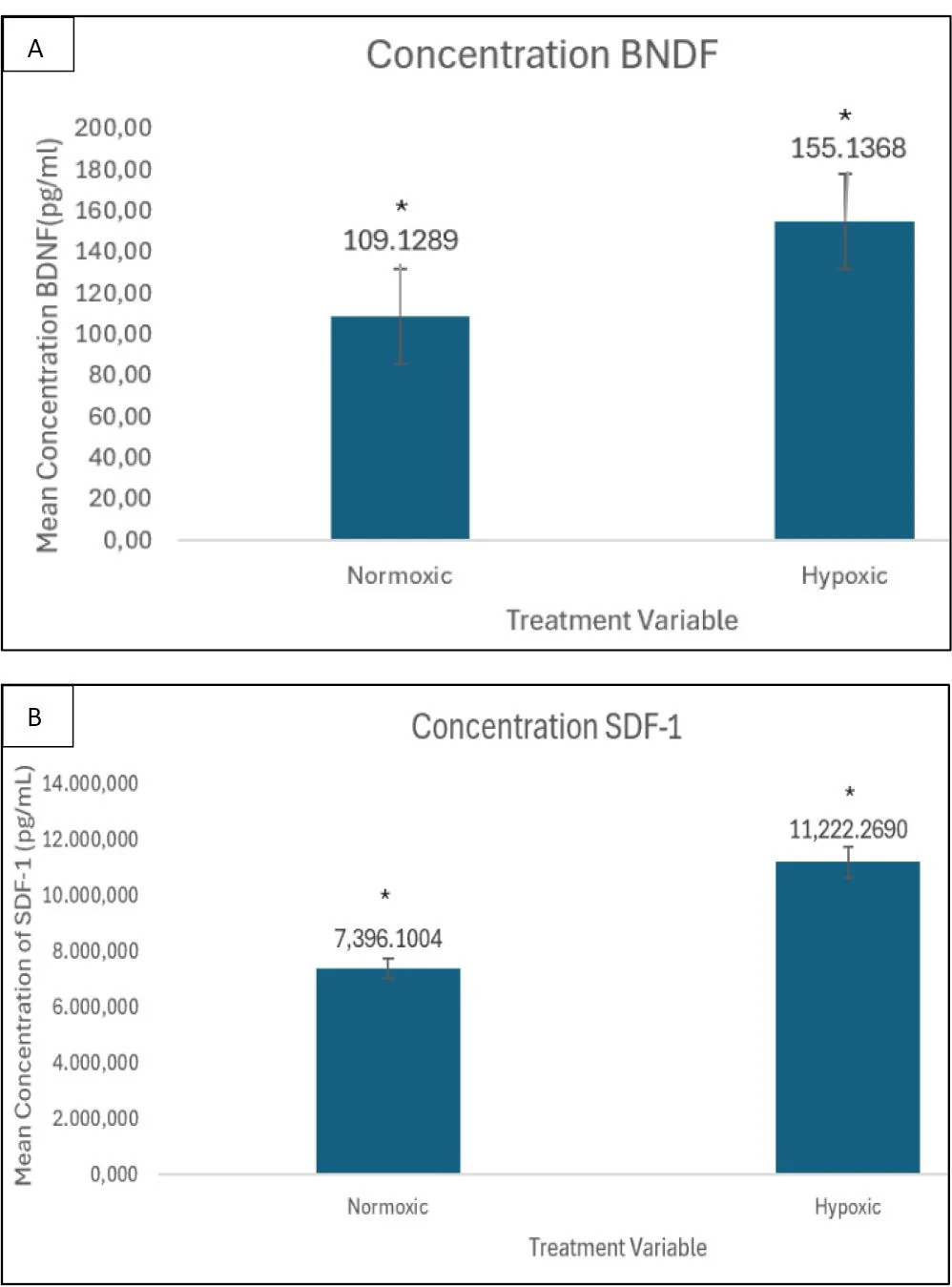
Levels of BDNF (A) and SDF-1 (B) in the secretome of *Macaca fascicularis* UC-MSCs after culture under hypoxic (3% O₂) or normoxic (21% O₂) conditions. Results are expressed as mean ± SD, with a significant difference between groups (*P* < 0.05)

###  SEM Analysis of UC-MSCs Exosome Morphology and Size Distribution

 UC-MSCs were seeded under normoxic and hypoxic conditions, and exosomes were isolated from serum-free media after 48 hours of incubation. SEM confirmed spherical exosomes with diameters < 100 nm (Figure 3).

**Figure 3:**
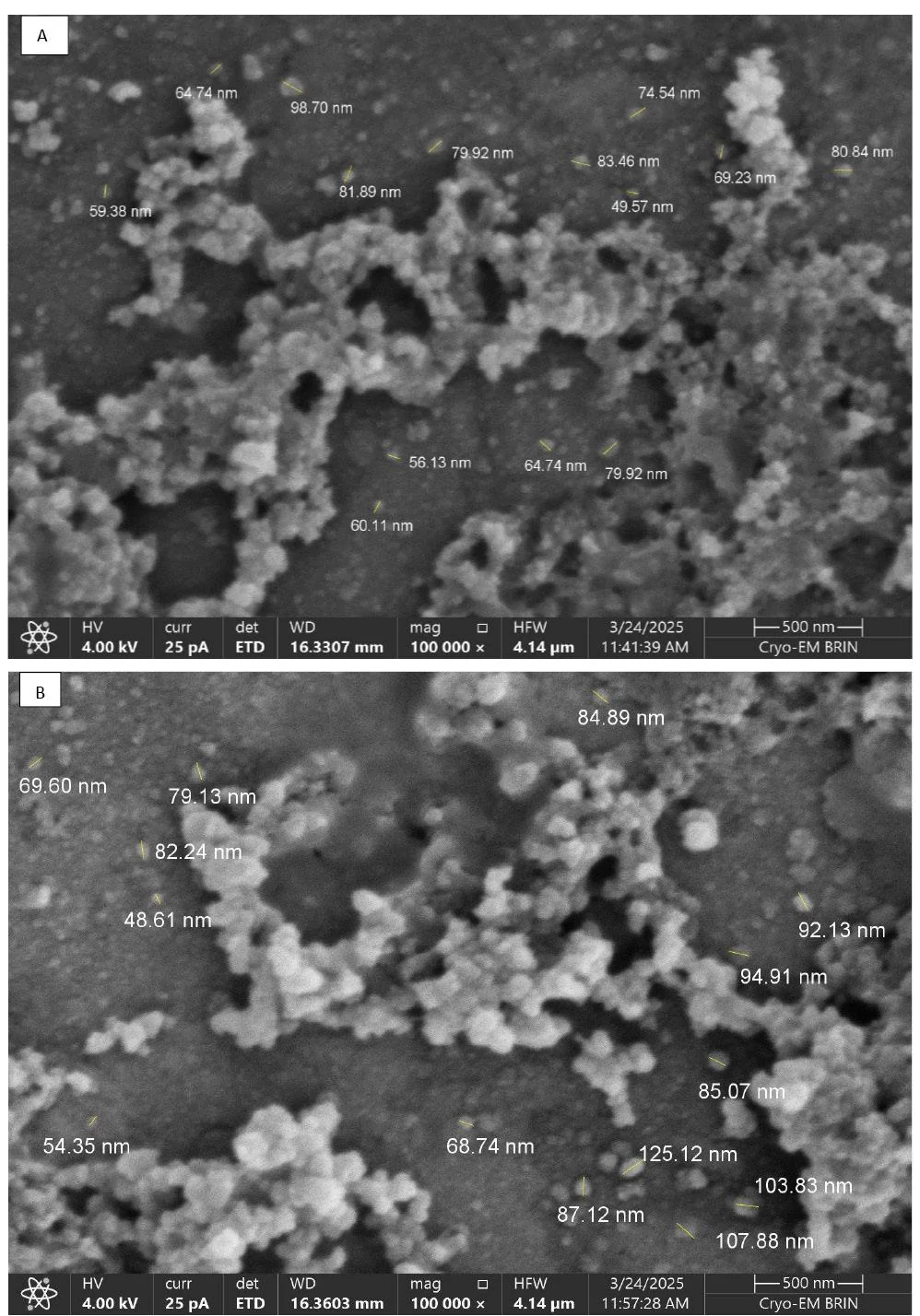
Secretome UC-MSCs SEM Normoxic (A), Secretome UC-MSCs SEM Hypoxia (B)

###  Immunocytochemical Characterization of SH-SY5Y Cells

 Immunocytochemistry for Nestin confirmed the neuroprogenitor status of SH-SY5Y cells before treatment. Basal morphology showed round to oval cell bodies lacking neurites, clustered growth, and non-polarized structures characteristic of undifferentiated neuroblastoma cells (Figure 4A). Strong cytoplasmic Nestin staining further verified the predominance of NPC-like cells (Figure 4B), indicating maintenance of an immature progenitor phenotype under standard culture without spontaneous neuronal differentiation.

**Figure 4:**
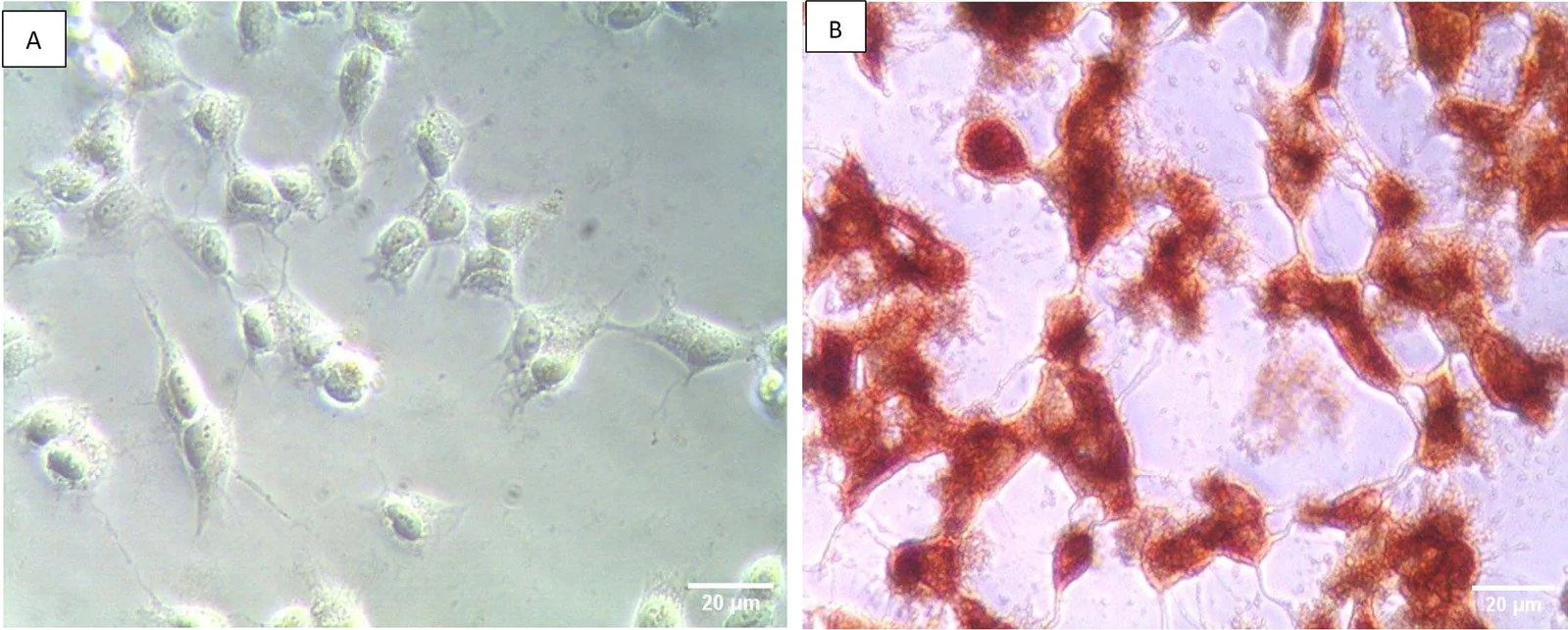
ICC Characterization Results: A. SH-SY5Y cells prior to immunocytochemistry (ICC). B. SH-SY5Y cells after ICC staining with Nestin marker

###  Culture of SH-SY5Y Cells Under OGD Conditions

 Undifferentiated SH-SY5Y cells exhibited typical neuroblast-like morphology, including high proliferation rates, rounded non-polar cell bodies, and short processes, with cells growing in dense, heterogeneous clusters comprising both epithelial-like and neuron-like cells (Figure 5A). Prior to treatment, cells adhered firmly and showed high viability (> 90%) with minimal neurite extension.

**Figure 5:**
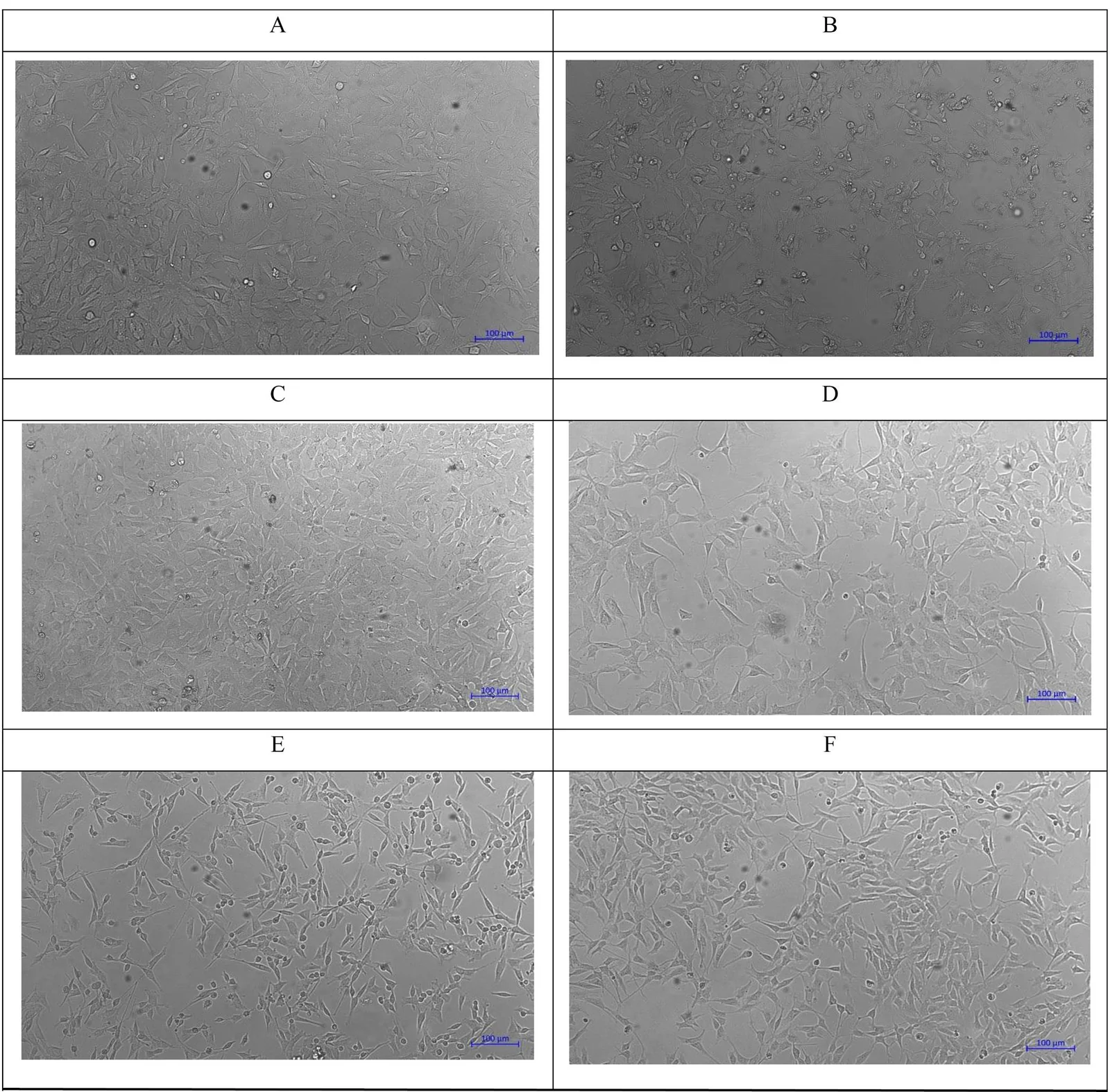
SH-SY5Y Cells Pre/Post-OGD (A).SHSY5Y Before OGD (B).After OGD (C).SH-SY5Y Control Cells (D). SH-SY5Y + OGD + DMEM with 10% FBS €. SH-SY5Y + OGD + Normoxic Secretome (F). SH-SY5Y + OGD + Hypoxic Secretome

 To model ischemic injury, SH-SY5Y cells underwent oxygen–glucose deprivation and reoxygenation (OGD/R), which caused rounding, neurite loss, vacuolization, and detachment (Figure 5B). Cells treated with DMEM + 10% FBS showed partial recovery but persistent stress, including neurite retraction and apoptosis (Figure 5D). Normoxia-derived secretome produced moderate improvement with extended neurites and better adhesion (Figure 5E). In contrast, hypoxia-conditioned UC-MSC secretome yielded the most pronounced protection, restoring neurite outgrowth, cytoskeletal integrity, and reducing apoptosis (Figure 5F). These results demonstrate that hypoxic priming enhances the reparative potential of UC-MSC secretome, promoting structural recovery and survival of ischemia-damaged neurons.

###  mRNA Expression Profile

 RT-qPCR showed that hypoxia-conditioned UC-MSC secretome significantly upregulated neurogenesis-related genes compared with all other groups. BDNF expression was highest in the hypoxia group (Figure 6A), while the normoxia group showed a smaller increase relative to DMEM + FBS and OGD controls.

**Figure 6:**
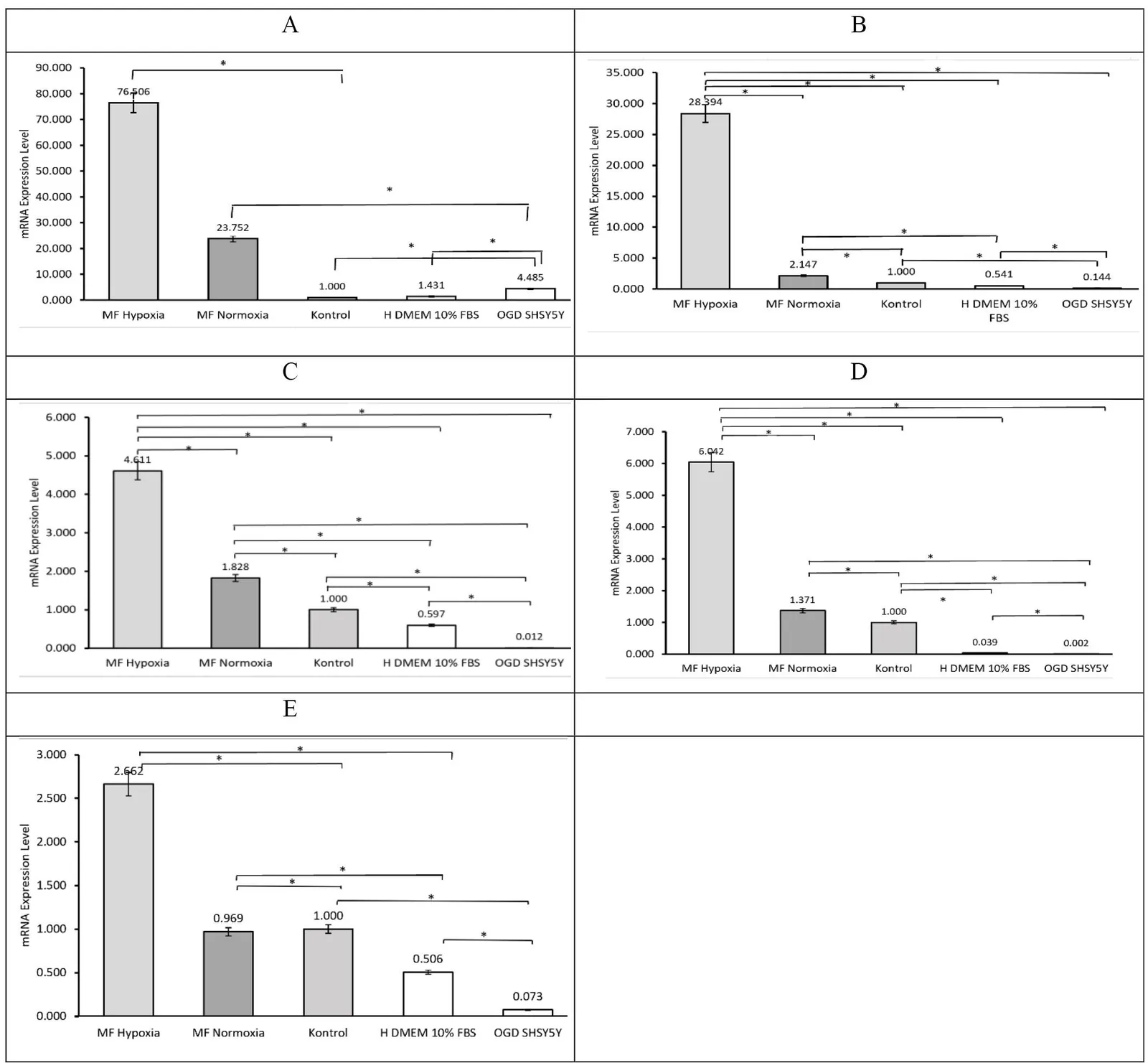
Relative mRNA expression of neurogenesis-related genes in SH-SY5Y cells following treatment with UC-MSC secretomes under normoxic or hypoxic (3% O₂) conditions. RT-qPCR analysis showed significant upregulation of (A) BDNF, (B) TrkB, (C) ERK1/2, (D) Nestin, and (E) β-tubulin in the hypoxia group compared with other treatments. Data are presented as mean ± SD; statistical significance was determined using one-way ANOVA with post hoc testing (*P* < 0.05)

 TrkB and ERK1/2, key mediators of neurotrophic signaling, were most strongly expressed in the hypoxia group, with lower levels in normoxia and controls (Figure 6B, C). Nestin was also markedly elevated under hypoxia, indicating preserved progenitor characteristics and a pro-neurogenic environment. β-tubulin, a marker of neuronal maturation, peaked in the hypoxia group and remained above controls in the normoxia group. Together, these findings confirm the superior neurogenic activity of hypoxia-conditioned secretome (Figure 6D, E).

## Discussion

 This study demonstrates that hypoxia-conditioned UC-MSC secretome significantly enhanced neuroprotective and neuroregenerative responses in an OGD/R stroke model. Hypoxic secretome (3% O₂) induced the greatest upregulation of BDNF, TrkB, ERK1/2, Nestin, and β-tubulin, which corresponded with improved SH-SY5Y viability, adhesion, and neurite outgrowth, highlighting the therapeutic advantage of hypoxic preconditioning for MSC-derived secretomes.^5^

*Macaca fascicularis* UC-MSCs exhibited typical MSC features, including spindle morphology, mesenchymal marker expression, and trilineage differentiation. Hypoxia (3% O₂) markedly increased secretion of BDNF and SDF-1 compared with normoxia and most reported MSC sources. BDNF levels were comparable to those of gingiva- and adipose-derived MSCs under moderate hypoxia but lower than Wharton’s Jelly MSCs at 2% O₂.^24–26^ In contrast, SDF-1 secretion was substantially higher than published values, suggesting potential species- or niche-specific enhancement.^18^

 Elevated BDNF and SDF-1 under hypoxia are highly relevant for stroke therapy, as SDF-1 recruits neural stem cells via CXCR4, while BDNF promotes their differentiation through TrkB–ERK signaling.^27–30^ Our data support these mechanisms, with significantly upregulated TrkB and ERK1/2 mRNA expression observed under hypoxic treatment. The activation of TrkB–MAPK and TrkB–PI3K/Akt pathways contributes to cell survival, synaptic plasticity, and anti-apoptotic signaling critical factors in recovery from ischemic injury.^31–36^

 Additionally, the maintenance of Nestin expression in SH-SY5Y cells post-treatment indicates preserved progenitor characteristics and potential for future differentiation.^37–39^ However, the absence of mature neuronal markers post-treatment suggests that additional differentiation cues (e.g., exogenous BDNF or NGF) are necessary for terminal maturation.^40,41^ Still, the increased β-tubulin expression under hypoxic conditions reflects early commitment toward neuronal lineage and cytoskeletal reorganization.

 Our ischemia/reperfusion model using 6-hour OGD followed by 24 hour reoxygenation successfully mimicked the neuronal injury cascade observed in stroke, including mitochondrial dysfunction, oxidative stress, and caspase activation leading to apoptosis.^42,43^ This model provided a reproducible platform for evaluating the neuroprotective effect of secretome therapies. Hypoxia-treated groups showed reversal of ischemic morphology, consistent with prior reports that MSC secretome mitigates damage via apoptotic and oxidative pathway modulation.^42,44,45^

 From a mechanistic standpoint, the BDNF-TrkB signaling axis remains central to therapeutic neurogenesis. TrkB activation triggers downstream ERK1/2 and PI3K/Akt pathways that support neuronal survival, axonal growth, and plasticity. The marked upregulation of ERK1/2 in this study corroborates findings from cerebral ischemia models, where ERK activation was linked to enhanced neurogenesis and cognitive recovery.^36,46,47^ Therefore, MSC-derived secretome, particularly when hypoxia primed, acts not only as a trophic reservoir but also as a molecular activator of intrinsic neuronal repair pathways.

 Furthermore, extracellular vesicles (EVs), including exosomes present in the secretome, contribute to paracrine signaling by transferring regulatory miRNAs, proteins, and lipids to target cells.^48–50^ These vesicles likely complement soluble factor activity, amplifying the reparative effects observed.

 While rodent MSC secretomes have shown consistent benefit in ischemic models, their predictive power for clinical translation is limited. Using Macaca fascicularis UC-MSCs provides a closer physiological and immunological match to humans, bridging the gap between rodent studies and clinical application.^16,17^ Our results show that primate secretome not only reproduces but also strengthens key neurogenic pathways (BDNF–TrkB–ERK), with notably higher SDF-1 secretion compared to rodent MSCs, underscoring its value as a meaningful preclinical platform.

 In conclusion, the present findings highlight the promising therapeutic potential of hypoxia-conditioned *Macaca fascicularis* UC-MSC secretome as a cell-free regenerative strategy to enhance neurogenesis and support neuronal recovery following ischemic injury. By enhancing key neurogenic and chemotactic signaling pathways (BDNF, SDF-1, TrkB, ERK1/2), this approach addresses critical challenges in stroke recovery. Future research should focus on refining preconditioning protocols, scaling secretome production, and validating in vivo efficacy for translational applications.

## Conclusion

 This study demonstrates that hypoxia preconditioned secretome derived from *Macaca fascicularis* UC-MSCs possesses superior neuroregenerative potential compared to its normoxia conditioned counterpart in an in vitro ischemic stroke model. Using the SH-SY5Y cell line subjected to OGD to mimic the penumbral region of ischemic injury, we confirmed the model’s validity through the induction of hypoxia-responsive phenotypes and the expression of neuronal progenitor marker Nestin. Notably, the hypoxic UC-MSC secretome significantly elevated levels of BDNF and SDF-1, and pronouncedly upregulated key neurogenesis-associated genes including BDNF, TrkB, ERK1/2, Nestin, and β-tubulin. In conclusion, hypoxia-conditioned UC-MSC secretome represents a promising cellular therapeutic candidate that enhances neurogenesis and may contribute to improved regenerative outcomes following ischemic stroke.

## Competing Interests

 The authors declare that they have no conflict of interest.

## Ethical Approval

 The study was conducted following animal welfare principles and has received ethical approval from the Biofarma Commission for Laboratory Animal Welfare and Use under ethical clearance number 01/IACUC_BF/X/22.
